# Monitoring bromide effect on radiolytic yields using *in situ* observations of uranyl oxide precipitation in the electron microscope†

**DOI:** 10.1039/c8ra01706a

**Published:** 2018-05-22

**Authors:** Edgar C. Buck, Richard S. Wittman, Chuck. Z. Soderquist, Bruce K. McNamara

**Affiliations:** Pacific Northwest National Laboratory 902 Battelle Blvd. Richland WA 99352 USA edgar.buck@pnnl.gov

## Abstract

During electron microscopy observations of uranium-bearing phases and solutions in a liquid cell, the electron beam induced radiolysis causes changes in the chemistry of the system. This could be useful for investigating accelerated alteration of UO_2_ and can be also used to monitor radiolytic effects. Low concentrations of bromide in aqueous solutions are known to reduce the generation rate of H_2_O_2_ during radiolysis and increase H_2_ production. We deduced the presence of radiolytic H_2_O_2_ by monitoring the formation of a uranyl peroxide solid from both solid UO_2_ and a solution of ammonium uranyl carbonate at neutral pH. Additionally, the effect of bromine on water radiolysis was investigated through chemical modelling and *in situ* electron microscopy. By measuring the contrast in the electron microscopy images it was possible to monitor H_2_O_2_ formation and diffusion from the irradiated zone in agreement with the models.

## Abstract 2

During electron microscopy observations of uranium-bearing phases and solutions in a liquid cell, the electron beam induced radiolysis causes changes in the chemistry of the system.

## Graphical abstract

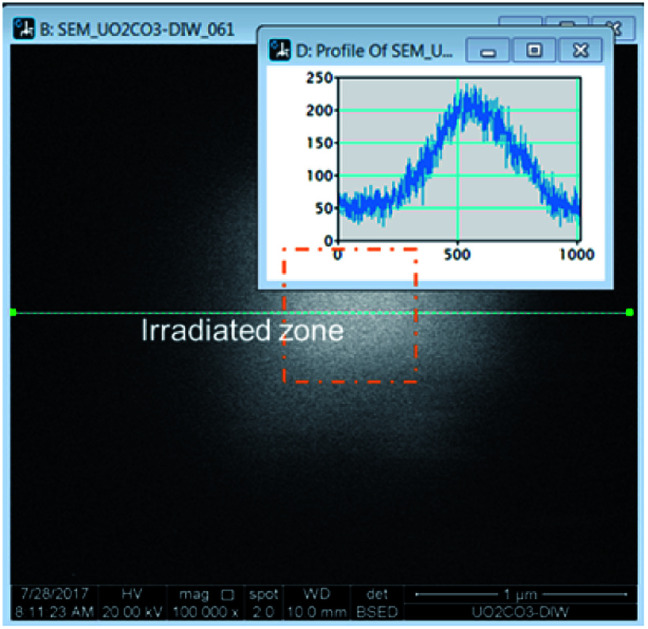

## Introduction

Electron-beam-induced processes that occur during liquid cell *in situ* electron microscopy can drive pH changes, reduce metal ions, through the generation of reactive radiolytic species.^1–4^ Along with ionization, the interaction of an energetic electron beam with water molecules will generate very short-lived (10^−15^ s) electronic excitations that favourably de-excite through intermediate atomic and molecular radicals. The reaction of these radicals with the surrounding aqueous environment occurs on the scale of 10^−9^ s, resulting in six dominant species (H_2_O_2_, H_2_, OH˙, H˙, e_aq_^−^, and H^+^).^5^ The production rate of these species is described by a ‘*G*-value’ that represents the number of species produced per 100 eV deposited in the irradiated solution within a small volume, termed the radiation spur. The *G*-value depends on the linear energy transfer (LET) of the ionizing particle travelling through the medium. As the species form, they will also diffuse rapidly through the fluid phase, undergoing other reactions. Oxygen which is not a direct radiolytic product is produced outside the radiation spur; however, its magnitude typically matches the production rate of the other molecular species (H_2_ and H_2_O_2_).

The radiolytic processes that occur during liquid cell (LC) electron microscopy (EM) and can lead to many chemical effects. It is important that any observations are also supported by other methods that do not create these reactive species or with an understanding of how radiolysis will affect the LC. Nevertheless, the direct study of electron beam effects can provide intriguing insight into the chemistry of a system and that may help to solve the complexities of mineral dissolution.^6–8^

## Materials and methods

Experiments were conducted with both solid and dissolved U, and a control study was performed with DI water and solutions containing bromide as described below. For the dissolved U experiments, an approximately 10 mM solution of ammonium uranyl carbonate (AUC), [(NH_4_)_4_UO_2_(CO_3_)_3_] (522.2 g mol^−1^) was prepared by dissolving 0.0529 g of the uranyl phase in 10 mL of DI water. AUC is an important component in the industrial conversion of UF_6_ to UO_2_. The bromine solutions were made as follows: 10^−2^ M solution of KBr (119.0 g mol^−1^) was produced by dissolving 0.0153 g of KBr in 10 mL of DI water. To make the 10^−4^ M KBr solution, 0.1 mL of the stock 10^−2^ M solution was diluted to 10 mL. 1 mL of this solution was diluted to 10 mL to yield the 10^−5^ M solution, and 1 mL of this solution was diluted to 10 mL to yield the 10^−6^ M solution.

For the experiments with solid material, a UO_2_ powder was used (<45 μm size sieved material) that was subsequently crushed between two glass slides to create *a* < 5 μm particles which were suspended in deionised (DI) water.

Experiments were conducted with 6 μL of solution pipetted into a fresh QX-102 WetSEM sample cell (QuantomiX, Rehovot, Israel). These were examined at 15 keV and with a magnification of 100 000× using an FEI (Thermo Fisher Scientific, Inc., Hillsboro, Oregon, USA) Quanta 250FEG scanning electron microscope. Irradiations were performed by rastering the beam over a 1 μm^2^ area and taking backscattered electron (BSE) images every few seconds.

**Fig. 1:**
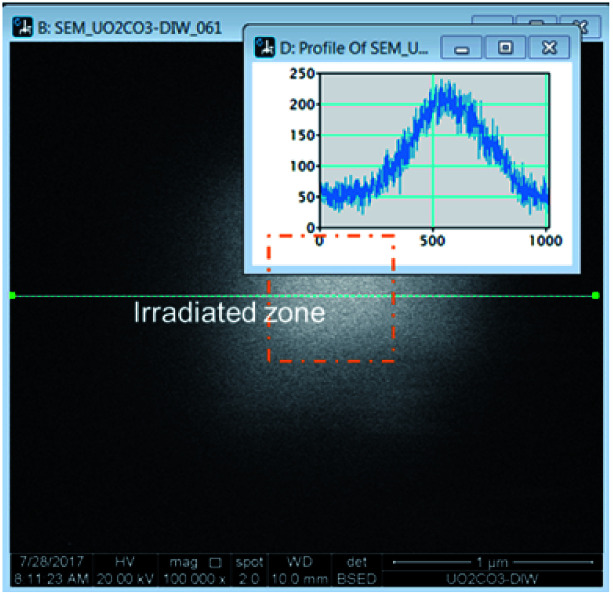
Method for determining the production of U-phase during irradiation.

The irradiation of the beam over a specific area could be accomplished in the microscope by selecting a specific area and allowing the beam to raster continuously. Over time, a white area would form, an image would be taken over a larger area encompassing a much larger area than was irradiated. The images revealed bright regions within the irradiated region. To determine the amount of material in these areas, a line histogram was generated as shown in Fig. 1. The integrated intensity yielded a value that was used to represent the amount of material precipitated. The brightness/contrast settings were kept constant during the irradiations. The background contrast level was the same in each experiment and this value was subtracted from the result to yield the integrated intensity from the presence of the peroxide phase.

A beam current of 10.5 pA was measured with Keithley Instruments (Tektronix Inc., Beaverton, Oregon, USA) 485 microvoltmeter. This was obtained with a spot size of 1.5 and the smallest available condenser aperture during both image collection and irradiation. This dose prevented rupture of the liquid cell window. The total dose rate was determined to be 7.28 e^−^ per nm^2^, assuming the irradiated region was 3 μm × 3 μm. As the electron beam was completely stopped within 5 μm, the estimated dose was 3.5 × 10^6^ Gy s^−1^.

The details of the measurement process are described in the ESI† section. Images were analysed with Gatan DigitalMicrograph 3.0 (Gatan Inc., Pleasanton, California, USA).^9^ A histogram of intensity was constructed from each image across the forming precipitates and was used as a means to measure H_2_O_2_ concentration. Nikon Elements 4.0 (Nikon Instruments, Inc., Melville, New York, USA) was used to determine the area of the features in the images.

Electron scattering effects were modelled using the CASINO program^10^ using the cross-section models by Browning *et al.*^11^ that were more applicable to the SEM beam energies.

### Radiolysis model

**Fig. 2:**
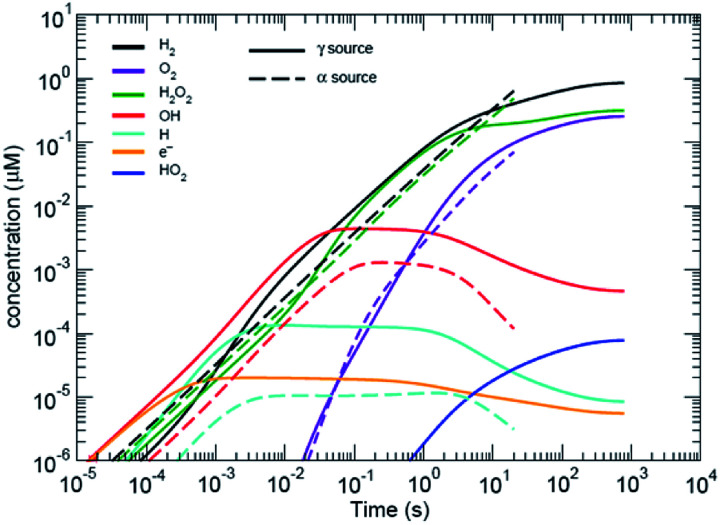
Predicted radiolytic species production rates for both α and β/γ radiation. The black lines corresponds to using the rate constants from ref. 15. α-Radiation results in a continuous production rate of H_2_ and H_2_O_2_; whereas, production under β,γ-radiation reaches a steady-state condition.

Water radiolysis generation models have been developed by Shoesmith and co-workers^12,13^ and Wittman *et al.*,^14^ for UO_2_ dissolution (see Fig. 2); and Schneider *et al.*,^4^ for modelling the effects of radiolysis during *in situ* LC-EM. All models use rate constants from Elliot and McCracken^15^ to solve a series of equations with stiff ordinary differential equation (ODE) solvers. The code by Wittman and co-workers^14^ uses the FORTRAN ODEPACK solvers developed at the Lawrence Livermore National Laboratory. The Schneider model uses MATLAB (MathWorks, Natick, Massachusetts, USA) solvers (ode15s) that are also based on the ODEPACK routines. The (Curtiss–Hirschfelder) stiff ODE solvers (MATLAB ode15s) are orders of magnitude faster than non-stiff (Dormand–Prince) solvers and MATLAB (ode45), for these types of problems that have orders of magnitude differences in many of the chemical rate equations to be solved. Like the Schneider model, the model used in this study also included the diffusion of species from the area of irradiation, but also included the effects of carbonate and halides.‡‡The radiolysis model is described in the ESI section and in several publically available reports referenced in the ESI.

**Fig. 3:**
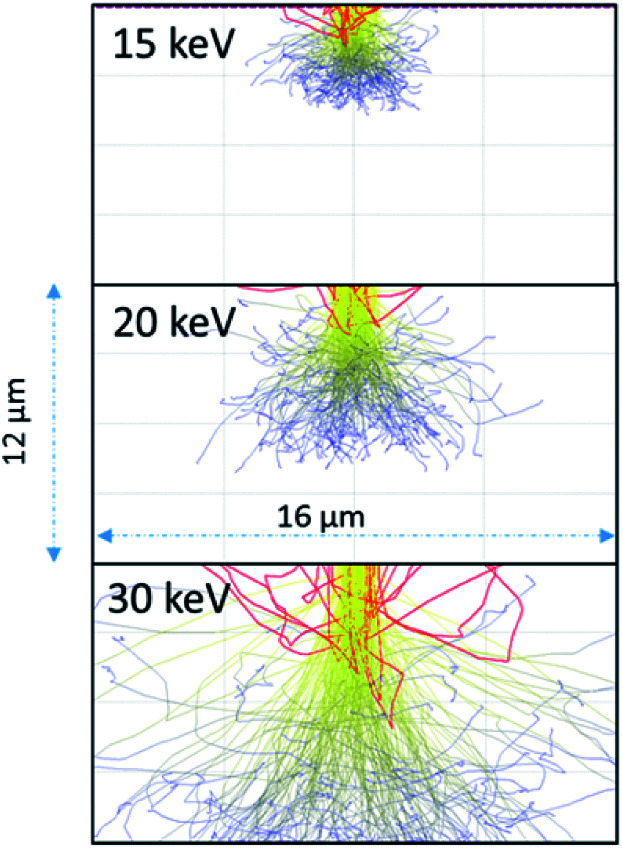
Anticipated electron scattering through water for a 1 μm wide beam at 15, 20, and 30 keV using the CASINO program.^10^ The red lines are backscattered electrons; the yellow are higher energy electrons leading to radiolytic events. The blue lines are scattered lower energy electrons although these electrons would still induce radiolytic events.

As the 15 keV electron beam is completely stopped within 3 μm of water (based on CASINO models (see Fig. 3)), the effective LET for 15 keV in this system is comparable to that β-particles from tritium (^3^H) where the *E*_max_ = 18.6 keV and the mean energy ∼ 5.7 keV. Butarbutar and co-workers have suggested that the effective high LET of ^3^H β-radiolysis results in *G*-values that are similar to that of neutrons.^16^ In this study, we adopted high LET *G*-values rather than those used for β/γ-radiation. All radiation spurs would occur within this small volume and, hence, molecular products would be favoured over radicals in this environment, as is the case with neutron or α-radiolysis. The *G*-values used in the model are listed in the ESI.† At higher keV there is greater scattering in regions outside of the 1 μm wide beam irradiated zone but this occurs deeper into the solution (see Fig. 3).

## Results of computational modelling

The coupled kinetics/diffusion rate equations for H_2_O_2_ on discrete special zones (*n*) can be expressed in terms concentrations [H_2_O_2_]_*n*_, fluxes *J*_*n*_ and dose rate 
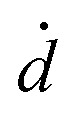
 according to:1
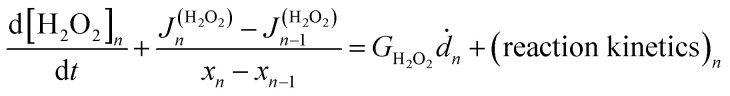


Assuming nonzero reaction kinetics and dose-rate only in the radiation zone (*x*_R_) with diffusion out to the boundary (*x*_B_), the steady-state solution to eqn (1) after inserting Fick's law fluxes containing diffusion constant *D* and boundary concentration [H_2_O_2_]_B_ can be written:2
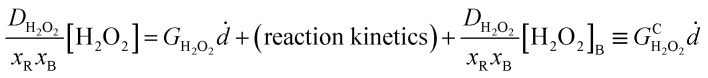


Eqn (2) serves as a working definition of a “conditional” *G*-value (*G*^c^) for [H_2_O_2_] which describes H_2_O_2_ generation conditional on the local water chemistry. As compared with the radiolysis model with all approximately 100 reactions to fully describe the system,^15^ we have shown that only 30–40 of the reactions are required to determine [H_2_O_2_] to one part in 10^−5^ and to preserve most of the predictions for the major species.^14^ This allows a systematic approach for model simplification and offers guidance in designing experiments for validation. To include the effects of bromide or iodide, several other [Br^−^] and [I^−^] related reactions need to be included.^17,18^

Table 1 includes the main reactions for predicting H_2_O_2_ generation in the presence of bromide (for ease of interpretation, we have adopted the same reaction numbers as those reported by Elliot and McCracken^15^). Without Br^−^ and for low O_2_ concentration, reaction 33 shows how the presence of H_2_ converts the ·OH˙ to the ·H˙ to accelerate H_2_O_2_ destruction, lowering its conditional *G*-value. At higher O_2_ concentrations, O_2_ effectively competes for ·H˙ radicals to disable H_2_O_2_ destruction (reaction 27).

**Table 1:** Important reactions included in the radiolysis model in this study

	Reaction	*K*_r_
3	H_2_O_2_ → H^+^ + ˙HO_2_^−^	1.1 × 10^−1^
4	H^+^ + ˙HO_2_^−^ → H_2_O_2_	5.0 × 10^10^
15	˙HO_2_ → O_2_^−^ + H^+^	1.3 × 10^6^
16	O_2_^−^ + H^+^ → ˙HO_2_	5.0 × 10^10^
23	e_aq_^−^ + H_2_O_2_ → ˙OH + OH^−^	1.1 × 10^10^
26	˙H + H_2_O_2_ → ˙OH + H_2_O	9.0 × 10^7^
27	˙H + O_2_ → ˙HO_2_	2.1 × 10^10^
33	˙OH + H_2_ → ˙H + H_2_O	4.3 × 10^7^
34	˙OH + H_2_O_2_ → ˙HO_2_ + H_2_O	2.7 × 10^7^
35	˙HO_2_ + O_2_^−^ → ˙HO_2_^−^ + O_2_	8.0 × 10^7^
36	H_2_O_2_ → ˙OH + ˙OH	2.5 × 10^−7^
94	Br^−^ + ˙OH → BrOH^−^	1.1 × 10^10^
96	Br^−^ + ˙H → BrH^−^	0.0 × 10^6^
102	Br^−^ + H_2_O_2_ → Br^−^ + O_2_^−^ + 2HO^+^	2.5 × 10^9^
138	BrOH^−^ → Br^−^ + ˙OH	3.0 × 10^7^
139	BrOH^−^ → ˙Br + OH^−^	4.2 × 10^6^

**Fig. 4:**
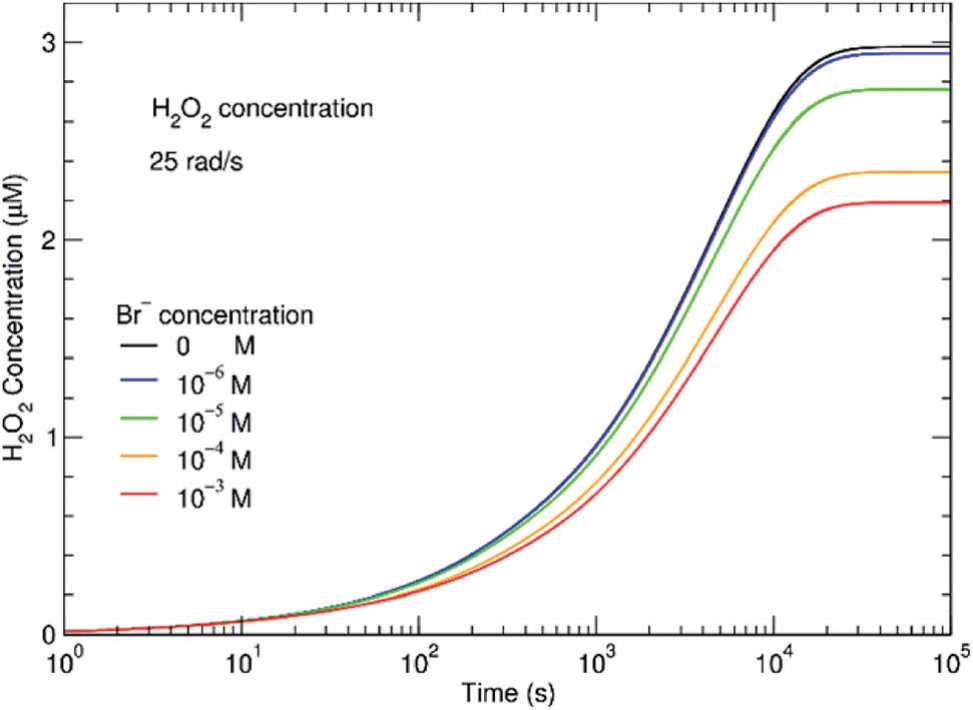
Effect of bromide on the steady-state concentration of H_2_O_2_ at a dose rate of 0.25 Gy s^−1^. Within minutes, the H_2_O_2_ concentration reaches a steady state concentration that depends on the initial Br^−^ added.

The effect of even small concentrations of Br^−^ can be seen in Fig. 4 where there is a decrease in the concentration of H_2_O_2_. As the H_2_O_2_ concentration decreases, H_2_ will increase. One question is whether this increasing H_2_ affects subsequent radiolysis. If the initial H_2_ concentration is high in the *in situ* LC, H_2_ would compete with Br^−^ for OH˙. The model indicates that even at an initial concentration of 100–400 μM of Br^−^, the presence of H_2_ should bring *G*^c^ from 0.1 to nearly 1 molecule/100 eV. In the experiments, we did not see evidence for this type of process and the results fit models where the initial concentration of H_2_ is low. During radiolysis, the secondary species, O_2_, is also generated. In these experiments, the solutions were in equilibrium with atmospheric O_2_. In the ESI,† the effect of initial concentrations of O_2_ is described. It is always important to consider the initial conditions. However, the models suggest that if the system was initially devoid of O_2_, there would be no difference in the H_2_ and H_2_O_2_ concentrations at steady state.

The radiation source is a surface or point, and allows for the radiolytic species to diffuse away from the irradiation region until steady-state conditions are reached distant from the source.^19^ This creates a different environment to a homogeneous field. In the electron microscope, the radiation source is also spatially isolated which may lead to different results compared to uniform irradiation.

The main reason for the effect is reaction 94 in Table 1. By competing for the ·OH˙, Br^−^ disables the mechanism for converting ·OH˙ to H˙ radical, strongly lowering the rate of destruction of H_2_O_2_ destruction.

## Monitoring bromide effect in the liquid cell

**Fig. 5:**
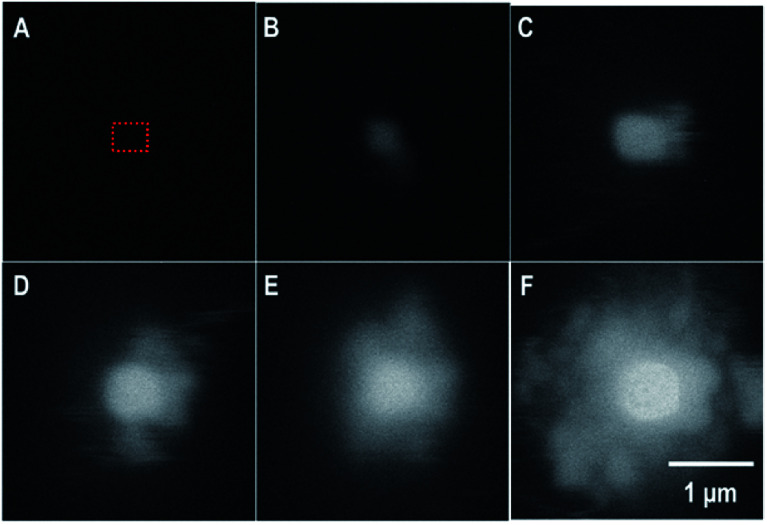
SEM-BSE images from the *in situ* LC showing the formation of a uranyl peroxide over time: (A) 0 s; (B) 78 s; (C) 194 s; (D) 314 s; (E) 479 s; and (F) 640 s. The irradiation occurred over *a* < 1 μm^2^ region in the center.§ The blurring is the result of the fast scan to capture the image without inducing excess irradiation to the area. The dotted red box represents the region that was irradiated.

In the *in situ* LC, the formation of a uranyl peroxide exhibited strong bright BSE contrast that was observed within seconds in the DI water case. Fig. 5 is§The method for determining contrast intensity of the images is discussed in the ESI. a series of SEM-BSE images that show the development of a uranyl peroxide precipitate in the central region where the irradiation was performed.

As the irradiation proceeded, material tended to precipitate far from the region of irradiation. This could be due to multiple scattering of the electrons leading to events outside the region irradiated directly or it could be from the diffusion of the longer-lived H_2_O_2_. We performed modelling of the electron scattering using the CASINO program^10^ to show the effects of different beam energies on electron scattering in the water. At 15 keV, the electrons are completely stopped within 3–5 μm. Resulting in an average LET close to 10 keV μm^−1^, similar to that of neutrons or tritium. As the beam energy is raised to 30 keV, the electrons penetrate much deeper and there is more scattering. The higher energy beam would be expected to have a lower overall LET, and would have radiolytic production distributions that were more similar to β/γ-sources. The effective of LET of electron beam sources is discussed further in the ESI.†

Similar time-resolved irradiations were performed with different concentrations of bromide (10^−4^ M, 10^−5^ M, and 10^−6^ M) with ammonium uranyl carbonate and monitored with BSE images. The integrated BSE contrast across the collected images was summed and plotted in Fig. 7. These results clearly show the dramatic effect of bromide on H_2_O_2_ production. As the bromide concentration was increased to 10^−4^ M, the bright contrast from the uranyl peroxide precipitate reduced in intensity significantly. Even at low bromide concentrations (10^−5^ M and 10^−6^ M) there was a slow-down in the production of H_2_O_2_ based on the reduction in the contrast from the U-peroxide phase.

**Fig. 6:**
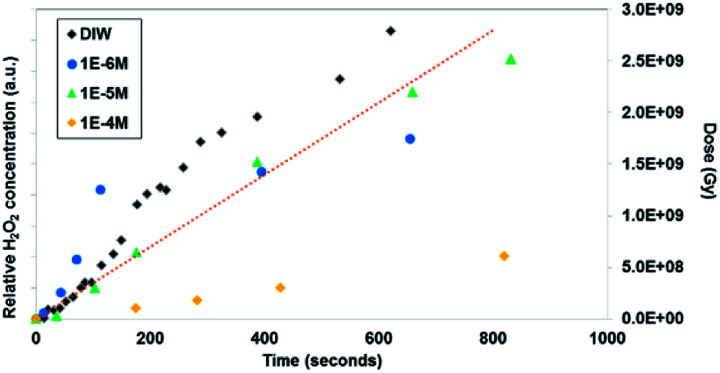
*In situ* microscopy measurement of uranyl peroxide formation with time and Br concentration (measurement method described in the ESI† section). The dotted line shows the dose (measured in Greys) estimated in the irradiated region with time.

**Fig. 7:**
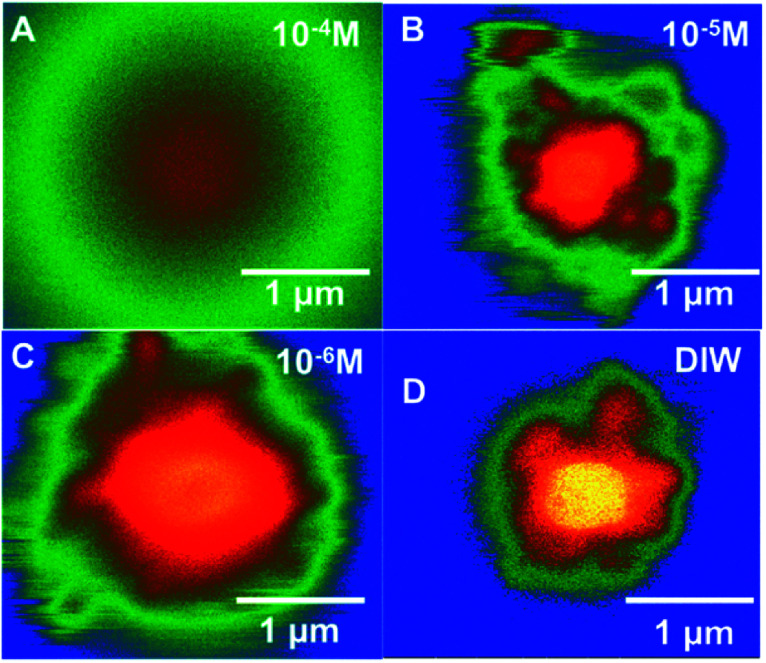
Colourized/heat mapped SEM-BSE images after 7 min of irradiation of ammonium uranyl carbonate in DI water and with three Br^−^ concentrations.

In Fig. 7, colourised/heat SEM-BSE images of the solutions with the different bromide solutions are all shown at approximately the same time of about 7 minutes of irradiation. The intensity of the image has been related to the concentration of U-peroxide. These images show, perhaps more clearly than the plot in Fig. 6, how the bromide influenced the precipitation phenomena. As Br^−^ increased, there was a reduction in the U-peroxide phase concentration. This is in agreement with the model where the steady state concentration of H_2_O_2_ is impacted by Br^−^.

The addition of milli-molar amounts of Br^−^ is commonly used to protect H_2_ from scavenging by OH radicals in reaction. The overall impact of Br^−^, as well as other halides (I^−^ and Cl^−^), is reaction with ˙OH radical (see below). The rate constants for this forward reaction with Br^−^ are faster than those for Cl^−^, at 1.1 × 10^10^ M^−1^ s^−1^ against 4.3 × 10^9^ M^−1^ s^−1^. However, the reverse reactions, are considerably slower for ˙BrOH^−^ (3.3 × 10^7^ M^−1^ s^−1^) than for ˙ClOH^−^ (4.3 × 10^9^ M^−1^ s^−1^).^20^˙OH + Br^−^ → ˙BrOH^−^˙BrOH^−^ → ˙Br + OH^−^˙BrOH^−^ + Br^−^ → ˙Br_2_^−^ + OH^−^

Additional reactions of the ˙Br^2−^ species, results in the formation of Br_3_^−^. Furthermore, additional Br radical species reactions in the radiolytic system tend to regenerate Br^−^. Altogether, these reactions result in Br^−^ becoming a much more potent scavenger of ˙OH radical even at much lower effective concentrations than Cl^−^. The e_aq_^−^ and ˙H will also react with Br_3_^−^ and ˙Br_2_^−^, resulting in the formation of H^+^ and Br^−^. Knowledge of the bromide concentration in contacting solutions is very important for understanding and predicting its potential impact on the radiolytic system. It is also possible that radioiodine from nuclear fission may be present in the wastes debris at ∼10^−4^ M. Experiments by Bauhn *et al.*^238^Pu-laced solutions containing solid UO_2_, do not agree with these results and models.^21^ Their results showed that 1 mM bromide-bearing solutions had no effect on H_2_O_2_ production.

**Fig. 8:**
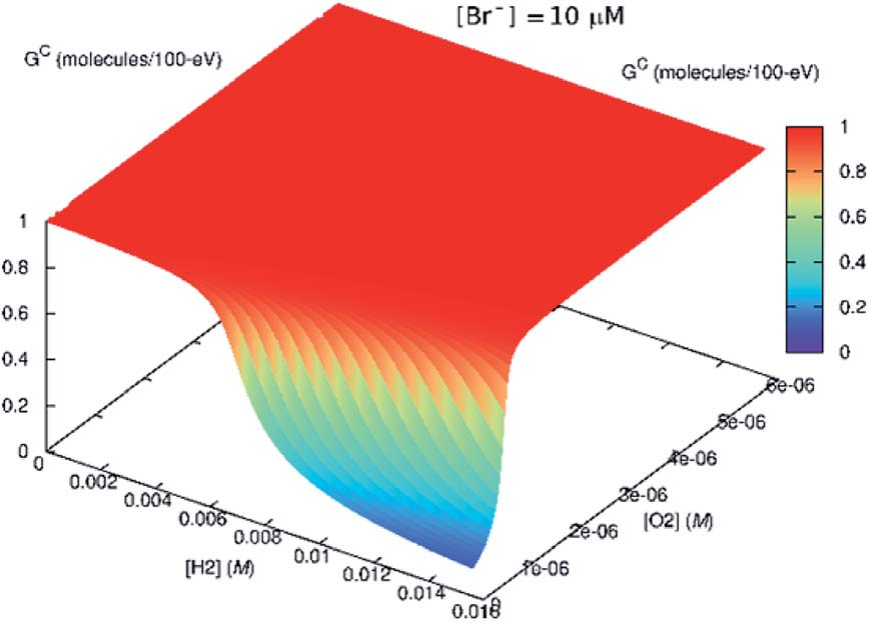
Effect of bromide ([Br^−^] = 10 μM) on the steady-state *G*^c^ value. If H_2_ is present in the system initially, the scavenging effect of Br^−^ diminishes.

According to our radiolytic model,‡ as the Br^−^ content in the solution increased, H_2_O_2_ production decreased and H_2_ production increased (see Fig. 8). In this instance, the radiation source is modelled as emerging from a surface, and the concentrations represent those distant from the radiation source, such that the solution is no longer directly exposed to α-particles.

This might imply that the building H_2_ concentration should affect further radiolysis and then prevent destruction of H_2_O_2_ despite the presence of bromide. In the *in situ* LC experiments, we do not see any delay production in H_2_O_2_. This is because there is no H_2_ at the start of the irradiation. If H_2_ had been present initially, this reversing effect, should have occurred.

## *In situ* oxidative corrosion of UO_2_

Dissolution of particles of UO_2_ was initially investigated by irradiating the UO_2_ particles in solution to establish the conditions for both preserving the cells and obtaining suitable images that could be analysed. Similar work has been performed by Traboulsi *et al.* using He^+^ ions^22^ and with direct addition of H_2_O_2_.^23^ These experiments resulted in the formation or *meta*-studtite from UO_2_ corrosion.

**Fig. 9:**
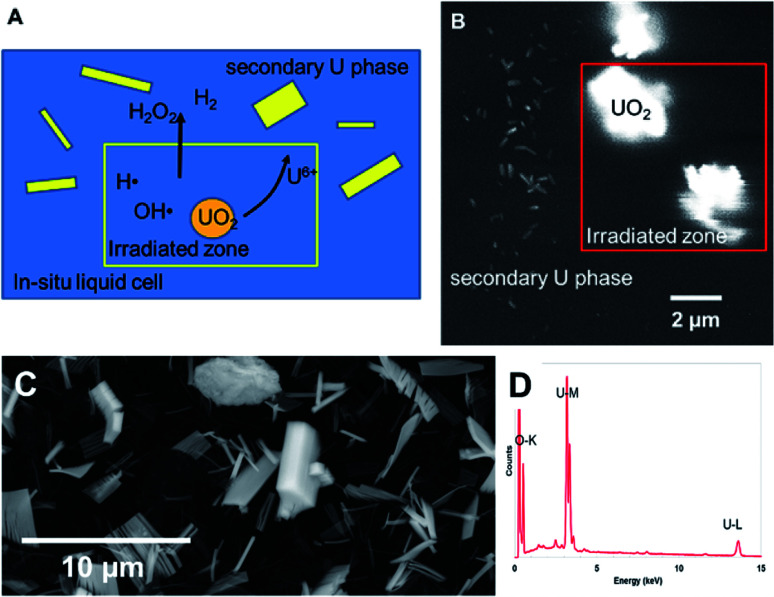
(A) Schematic showing the effect of localised electron irradiation on a solution containing UO_2_ particles. (B) SEM-BSE image showing the formation of studtite during irradiation of UO_2_ particles in solution. The irradiated zone is marked within the red outlined box. (C) SEM image of studtite formed from the alteration of irradiated U fuel in the K-Basins at the Hanford site, Washington, USA. (D) X-Ray energy dispersive analysis of the phase.

In Fig. 9, UO_2_ particles in DI water were irradiated continuously over a fixed area (indicated by the red square in Fig. 9b). The production of H_2_O_2_ from irradiation resulted in the dissolution of a portion of the UO_2_ and the precipitation of a uranyl oxide phase outside of the irradiated region over the course of several minutes. No significant changes were noted in the size of the initial UO_2_ particles because of their relatively large mass. Although it was not possible to identify directly the phase of the newly-formed uranium-bearing particles, it is likely (based on particle morphology and knowledge of H_2_O_2_ reaction with U) that these were the uranyl oxide peroxide phase studtite. The location of the studtite phase outside of the irradiated region points to the diffusion of H_2_O_2_ and the importance of considering this effect during irradiation of solutions in the electron microscope cells. The CASINO electron scattering model for 15 keV electrons shows that very little electron scattering occurs >3 μm outside the irradiated region (see Fig. 3), supporting the contention that the material found outside the irradiated region is from the diffusion of H_2_O_2_ and resulting reaction with U in solution.

Nuclear materials, when contacted with water, can induce radiolysis in the surrounding solution from their radioactive emissions. Uraninite (UO_2_) is thermodynamically unstable under oxidizing conditions and combined with the alpha (α)-radiation field drive oxidative dissolution.^24–27^ The oxidation process is controlled by the concentration of H_2_O_2_; where the high oxidation potential will lead to the formation of soluble UO_2(aq)_^2+^.^28^ In the presence of high concentrations of H_2_O_2_, a secondary phase studtite [(UO_2_)(O_2_)(H_2_O)_2_·2H_2_O] may precipitate.^29–32^ Indeed, studtite has been identified on the surface of spent UO_2_ nuclear fuel.^33–36^ Studtite is known to be stable in H_2_O_2_ containing environments.^37^ The recent studies by Bauhn *et al.* that show 1 mM bromide did not impact H_2_O_2_ production in an alpha-dominated environment^21^ may be due the spatial location of the radiation field. We show that studtite was formed outside of the irradiated region in agreement with the findings of Traboulsi *et al.*^22^ for He^+^ ions. It is unknown if direct irradiation would similarly allow the crystallisation of the secondary phase.

## Conclusions

*In situ* LC electron microscopy was used to examine effect of different bromide concentrations on the production of H_2_O_2_ by monitoring precipitation of a U-peroxide phases during the irradiation. We were able to corroborate a radiolysis model for H_2_O_2_ generation by observing a reduction in H_2_O_2_ concentration with added bromide; although, this is in disagreement with the work of Bauhn *et al.*^21^ The study also points to the role of radiolysis in the electron microscope that can easily overwhelm a chemical system. However, with careful selection of beam current, beam energy, and dose, it is possible to conduct useful experiments with these types of liquid cell. We have shown that although it is possible to investigate radiolytic phenomena with the *in situ* LC, models need to include the diffusion of species to account for the nature of species migration in the cell and not just the steady state conditions under homogeneous irradiation.

## Conflicts of interest

There are no conflicts to declare.

## Supplementary Material

RA-008-C8RA01706A-s001

## References

[cit1] de Jonge N., Ross F. M. (2011). Electron microscopy of specimens in liquid. Nat. Nanotechnol..

[cit2] Evans J. E. (2011). *et al.*, Controlled Growth of Nanoparticles from Solution with In Situ Liquid Transmission Electron Microscopy. Nano Lett..

[cit3] Abellan P. (2017). *et al.*, The formation of cerium(iii) hydroxide nanoparticles by a radiation mediated increase in local pH. RSC Adv..

[cit4] Schneider N. M. (2014). *et al.*, Electron–Water Interactions and Implications for Liquid Cell Electron Microscopy. J. Phys. Chem. Lett..

[cit5] Garrett B. C. (2005). The role of water in electron-initiated processes and radical chemistry: issues and scientific advances. Chem. Rev..

[cit6] De Yoreo J. J. (2015). *et al.*, Crystallization by particle attachment in synthetic, biogenic, and geologic environments. Science.

[cit7] Li D. (2012). *et al.*, Direction-Specific Interactions Control Crystal Growth by Oriented Attachment. Science.

[cit8] Woehl T. J. (2012). *et al.*, Direct in situ determination of the mechanisms controlling nanoparticle nucleation and growth. ACS Nano.

[cit9] Mitchell D. R. G., Schaffer B. (2005). Scripting-customised microscopy tools for Digital Micrograph™. Ultramicroscopy.

[cit10] Hovington P., Drouin D., Gauvin R. (1997). CASINO: a new monte carlo code in C language for electron beam interaction—part I: description of the program. Scanning.

[cit11] Browning R. (1995). *et al.*, Low-energy electron/atom elastic scattering cross sections from 0.1–30 keV. Scanning.

[cit12] Shoesmith D. W., Sunder S. (1992). The prediction of nuclear fuel (UO_2_) dissolution rates under waste disposal conditions. J. Nucl. Mater..

[cit13] Liu N. (2017). *et al.*, Modelling the radiolytic corrosion of α-doped UO_2_ and spent nuclear fuel. J. Nucl. Mater..

[cit14] Wittman R. S. (2014). *et al.*, Conditions for critical effects in the mass action kinetics equations for water radiolysis. J. Phys. Chem. A.

[cit15] Elliot A. J., McCracken D. R. (1990). Computer modelling of the radiolysis in an aqueous lithium salt blanket: Suppression of radiolysis by addition of hydrogen. Fusion Eng. Des..

[cit16] Butarbutar S. L. (2014). *et al.*, Self-radiolysis of tritiated water. 2. Density dependence of the yields of primary species formed in the radiolysis of supercritical water by tritium [small beta]-particles at 400 [degree]C. RSC Adv..

[cit17] Mustaree S. (2014). *et al.*, Self-radiolysis of tritiated water. 3. The [radical dot]OH scavenging effect of bromide ions on the yield of H_2_O_2_ in the radiolysis of water by 60Co [gamma]-rays and tritium [small beta]-particles at room temperature. RSC Adv..

[cit18] Buxton G. V., Mulazzani Q. G. (2007). On the hydrolysis of iodine in alkaline solution: a radiation chemical study. Radiat. Phys. Chem..

[cit19] Nielsen F., Lundahl K., Jonsson M. (2008). Simulations of H_2_O_2_ concentration profiles in the water surrounding spent nuclear fuel. J. Nucl. Mater..

[cit20] LaVerne J. A., Ryan M. R., Mu T. (2009). Hydrogen production in the radiolysis of bromide solutions. Radiat. Phys. Chem..

[cit21] Bauhn L. (2017). *et al.*, The Effect of Bromide on Oxygen Yields in Homogeneous α-Radiolysis. MRS Adv..

[cit22] Traboulsi A. (2015). *et al.*, Radiolytic corrosion of uranium dioxide induced by He^2+^ localized irradiation of water: role of the produced H_2_O_2_ distance. J. Nucl. Mater..

[cit23] Clarens F. (2004). *et al.*, Formation of Studtite during the Oxidative Dissolution of UO_2_ by Hydrogen Peroxide: A SFM Study. Environ. Sci. Technol..

[cit24] Wronkiewicz D. J. (1996). *et al.*, Ten-year results from unsaturated drip tests with UO_2_ at 90 °C: implications for the corrosion of spent nuclear
fuel. J. Nucl. Mater..

[cit25] Ewing R. C. (2015). Long-term storage of spent nuclear fuel. Nat. Mater..

[cit26] Wronkiewicz D. J., Buck E. (1999). Uranium mineralogy and the geologic disposal of spent nuclear fuel. Rev. Mineral. Geochem..

[cit27] Amme M. (2012). *et al.*, Combined effects of Fe(ii) and oxidizing radiolysis products on UO_2_ and PuO_2_ dissolution in a system containing solid UO_2_ and PuO_2_. J. Nucl. Mater..

[cit28] Grenthe I. , *et al.*, Uranium, in The chemistry of the actinide and transactinide elements, Springer Netherlands, 2008, pp. 253–698

[cit29] Burns P. C., Hughes K.-A. (2003). Studtite, [(UO_2_)(O_2_)(H_2_O)_2_](H_2_O)_2_: the first structure of a peroxide mineral. Am. Mineral..

[cit30] Kubatko K.-A. H. (2003). *et al.*, Stability of Peroxide-Containing Uranyl Minerals. Science.

[cit31] Weck P. F., Kim E., Buck E. C. (2015). On the mechanical stability of uranyl peroxide hydrates: implications for nuclear fuel degradation. RSC Adv..

[cit32] Mallon C. (2012). *et al.*, Physical Characterization and Reactivity of the Uranyl Peroxide [UO_2_(η_2_-O_2_)(H_2_O)_2_]·2H_2_O: Implications for Storage of Spent Nuclear Fuels. Inorg. Chem..

[cit33] Hanson B. D. (2005). *et al.*, Corrosion of commercial spent nuclear fuel. 1. Formation of studtite and metastudtite. Radiochim. Acta.

[cit34] McNamara B. (2005). *et al.*, Corrosion of commercial spent nuclear fuel. 2. Radiochemical analyses of metastudtite and leachates. Radiochim. Acta.

[cit35] Forbes T. Z. (2011). *et al.*, Alteration of dehydrated schoepite and soddyite to studtite, (UO_2_)(O-2)(H_2_O)_(2)_(H_2_O)_(2)_. Am. Mineral..

[cit36] Armstrong C. R. (2012). *et al.*, Uranyl peroxide enhanced nuclear fuel corrosion in seawater. Proc. Natl. Acad. Sci. U. S. A..

[cit37] Sattonnay G. (2001). *et al.*, Alpha-radiolysis effects on UO_2_ alteration in water. J. Nucl. Mater..

