# Supplementary material for: Monitoring bromide effect on radiolytic yields using in situ observations of uranyl oxide precipitation in the electron microscope
Source: RSC Adv. 2018 May 22;8(33):18227–33. doi: 10.1039/c8ra01706a (PMC9080531; doi:10.1039/c8ra01706a)
Supplement: RA-008-C8RA01706A-s001 [file RA-008-C8RA01706A-s001.pdf]

Electronic Supplementary Material (ESI) for RSC Advances.
This journal is © The Royal Society of Chemistry 2018

Supplementary Information

Edgar C. Buck,* Rick S. Wittman, Chuck Z. Soderquist, Bruce McNamara,

Energy and Environment Directorate, Pacific Northwest National Laboratory, 902 Battelle Blvd., Richland, WA 99352

In-situ Observations

We used *in-situ* liquid cells (LC) in a scanning electron microscope (SEM) using backscattered electron (BSE) contrast to allow the direct observation of the precipitation and dissolution of uranium (U). Because of the very high BSE contrast from the U-bearing phases, it was possible to determine growth/dissolution rate with high spatial resolution and investigate the dynamics of the process. In **Figure S1**, particles of UO_2 were irradiated over the entire image area for a period of time. Here, the beam was on continuously, generating radiolytic species in solution. If H_2O_2 was generated by the electron beam, it would be predicted that the UO_2 would partially dissolve and eventually, a U-peroxide phase would precipitate. After a period of time (>10 minutes), a phase appeared on the surface of the UO_2 particles. Alteration of UO_2 with H_2O_2 is known to generate the secondary phase, studtite. This was confirmation that the electron beam was generating peroxide in sufficient concentration to induce both UO_2 oxidative dissolution and precipitation of a secondary phase. This was the first time that this had been observed in a wetSEM liquid cell although similar in-situ dissolution experiments have been performed with ex-situ radiation sources [1-3] and He^+ irradiation [4].

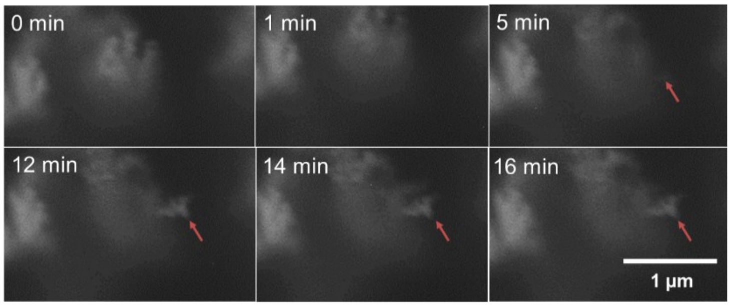

Figure S1 Electron beam irradiation inducing the formation of an alteration phase on the surface of UO_2. Example of in-situ EM generating H_2O_2 and causing the formation of a uranium phase.

The irradiation of the beam over a specific area could be accomplished in the microscope by selecting a specific area and allowing the beam to raster continuously. Over time, a white area would form, an image would be taken over an larger area encompassing a much larger area than was irradiated. The images revealed bright regions within the irradiated region. To determine the amount of material in these areas, a line histogram was generated. The intensity yielded a value that was used to represent the amount of material precipitated. The brightness/contrast settings were kept constant during the irradiations. The background contrast level was the same in each experiment and this value was subtracted from the result to yield the intensity from the presence of the peroxide phase.

H_2 Gas production measured in-situ

Three chemical reactions are considered responsible for the majority of the formation of molecular hydrogen during radiolysis:

$H + H \rightarrow H_2$	$k_1 = 7.8 \times 10^9\ M^{-1}s^{-1}$
$e_{aq}^- + e_{aq}^-\ (+2H_2O) \rightarrow H_2 + 2OH^-$	$k_2 = 5.5 \times 10^9\ M^{-1}s^{-1}$
$e_{aq}^- + H\ (+H_2O) \rightarrow H_2 + OH^-$	$k_3 = 2.5 \times 10^9\ M^{-1}s^{-1}$

The formation of H_2 could be monitored in the electron microscope with DIW (see **Figure S2**). The steady growth in the gas bubble consistent with the predictions.

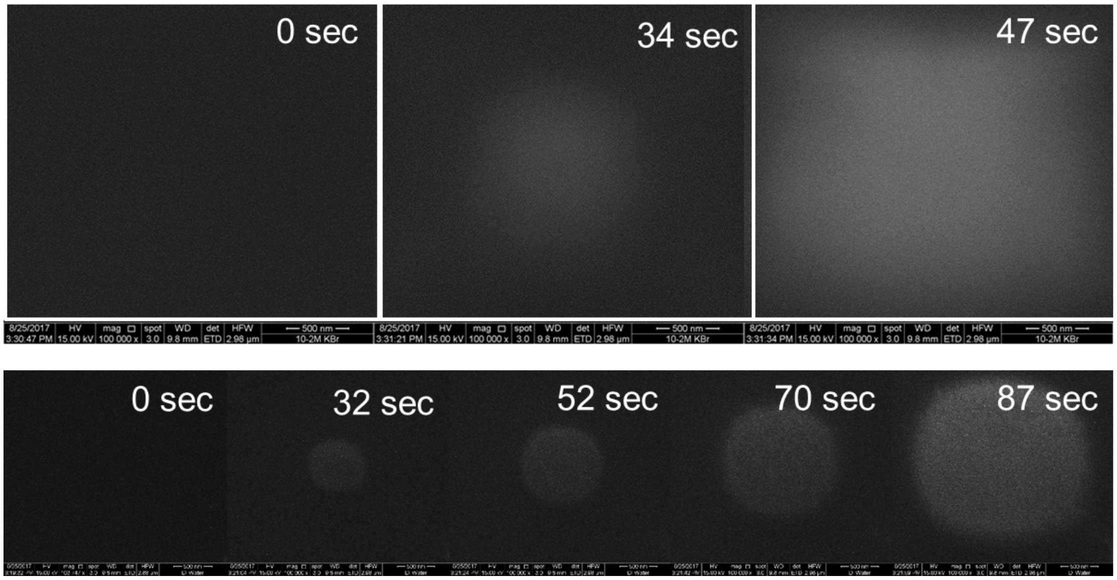

Figure S2 Irradiation of DIW (A) 0 sec, (B) 32 sec, (C) 52 sec, (D) 70 sec, (E) 87 sec

Radiolytic models confirm that the starting concentration of O_2 has little impact on radiolysis

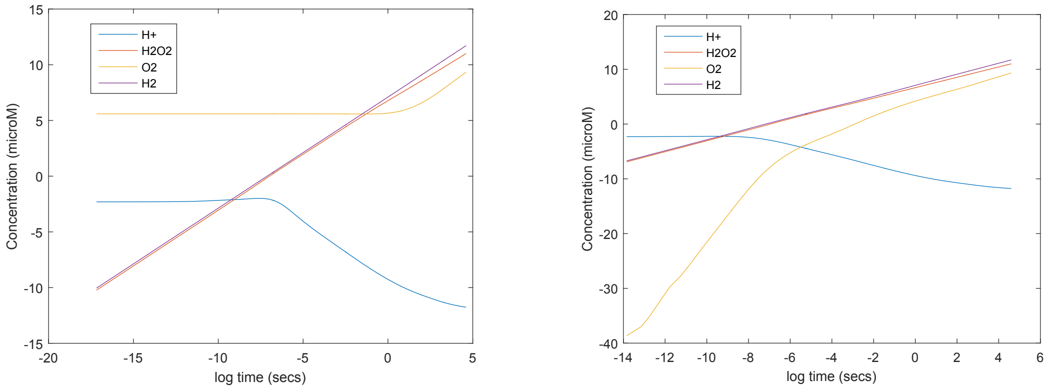

Figure S3 (A) In the presence of oxygen and (B) under anoxic conditions, the production rate of H_2 and H_2O_2 is invariant. Note the scales are different but the slopes are the same.

The starting concentration of O_2 has little impact on the overall generation of H_2O_2 and H_2 in the wetSEM cell. High LET radiolysis, results in the continuous generation of the molecular species with time (see **Figure S3**). This eventually results in sufficient gas generation that can rupture the cells. In contrast, low LET (higher energy electrons) would result in a limited concentration of these species as the high production rate of radicals prevents continuous gas production.

Radiolytic Chemical Modelling

Along with ionization, the interaction of energetic radiation with water molecules generates very short-lived (10^{-15} s) electronic excitations that de-excite through intermediate atomic and molecular radicals as shown in **Figure S4**. The reaction of these radicals with the surrounding aqueous environment occurs on the scale of 10^{-9} s, resulting in several dominant species—both stable and unstable. The G-values are strongly affected by radiation quality [or linear energy transfer (LET)]. In general, alpha particles have high LET and beta/gamma has low LET (see **Table S1**). However, many low-energy beta particles have high effective LET.

The electron microscope represents a special case for modeling radiation chemistry because of the nature of the rastering beam and the spatial confinement of the system in the z-direction. We modelled the behaviour of the beam at several different energies. At 15 keV, all radiolytic events take place within a small region leading to an effective high linear energy transfer. A similar idea was developed from a study of radiolysis from tritium [5, 6]. According to the computer program CASINO the 15 keV electron beam is almost completely stopped in water within 3 µm.

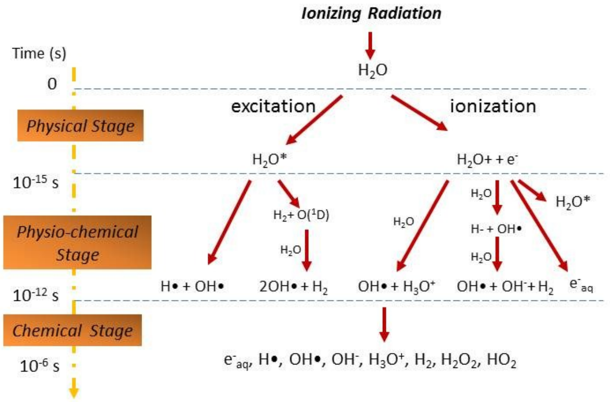

Figure S4. Schematic of reaction pathways in the radiolysis model (adapted from [7-10])

Table S1. Alpha particle (5 MeV) *G*-values

Species	*G*-value (molecules/100-eV
H^+	0.18
H_2O	–2.58
H_2O_2	1.00
e^-	0.15
·H	0.10
·OH	0.35
$\cdot HO_2$	0.10
H_2	1.20

This means that the linear energy transfer (LET) is large compared to γ- or x-rays and the subsequent g-values will be more like neutron or α-radiation values than high energy β/γ. Hence, we adopted G-values for high LET radiation, such as neutrons or α-particles to represent the behavior of electron irradiation in the wetSEM cell (see **Table S1** for the G-values used).

Table S2. Diffusion constants D_i

Species	e^-	$\cdot OH$	O_2^-	H_2O_2	O_2	H_2	Others
D_i [10^{-5} cm^2-s^{-1}]	4.9	2.3	1.5	1.9	2.5	6.0	1.5
$\Delta[H_2O_2]/[H_2O_2]$	$1x10^{-7}$	$5x10^{-5}$	0.0052	–25.5	$-1x10^{-3}$	–0.275	----

Along with ionization, the interaction of energetic radiation with water molecules can generate very short-lived (10^{-15} s) electronic excitations that favorably de-excite through intermediate atomic and molecular radicals. The reaction of these radicals with the surrounding aqueous environment occurs on the scale of 10^{-9} s, resulting in several dominant species – both stable and unstable. We take the conventional approach in representing the radiolyically-generated species at the later time scale with effective *G*-values. The *G*-values account for the effective fraction of radiative energy that contributes to the formation energy of the dominant radiolytic species. The products of *G*-values with the dose rate act as source terms to the kinetics equations for each for the species.

The coupled kinetics rate equations for the component concentrations $[A_i]_n$ are

$$\frac{d\,[A_i]_n}{dt} + \frac{J_n^{(i)} - J_{n-1}^{(i)}}{x_n - x_{n-1}} = G_i \dot{d}_n + \sum_{r=1}^{N_r} k_{ir} \prod_{j_r=1}^{n_r} [A_{j_r}]_n^{O_{jr}} \tag{1}$$

with rate constants k_{ir}, dose rate $\dot{d}_n$ and radiolytic generation constants G_i, where the diffusive currents ($J^{(i)}$) and diffusion constants (D_i) appear in the discretized Fick's Law according to

$$J_n^{(i)} = -2D_i \frac{[A_i]_{n+1} - [A_i]_n}{x_{n+1} - x_{n-1}} \tag{2}$$

for each component *i* in region *n*. **Table S2** shows the values of diffusion constants used in the model. For brevity, the "sum-of-products" on right-hand side of Eq. 1 expresses the sum of the product of reactant concentrations entering with reaction order O_{jr} where the multiplication-index j_r is over the n_r reactants for reaction *r*. The notation includes the final state order of component *i* produced by writing the rate constants k_{ir}, dependent on index *i*. That dependence amounts to an integer (which could be zero) multiplied by the reaction rate constants reported by Buck and Wittman [12] and Buck et al. [13].

References

1. Bailey, M.G., L.H. Johnson, and D.W. Shoesmith, *The effects of the alpha-radiolysis of water on the corrosion of uo2.* Corrosion Science, 1985. **25**(4): p. 233-238.
2. Jégou, C., et al., *Effect of external gamma irradiation on dissolution of the spent UO2 fuel matrix.* Journal of Nuclear Materials, 2005. **341**(1): p. 62-82.
3. Sattonnay, G., et al., *Alpha-radiolysis effects on UO2 alteration in water.* Journal of Nuclear Materials, 2001. **288**(1): p. 11-19.
4. Traboulsi, A., et al., *Radiolytic corrosion of uranium dioxide induced by He2+ localized irradiation of water: Role of the produced H2O2 distance.* Journal of Nuclear Materials, 2015. **467, Part 2**: p. 832-839.
5. Mustaree, S., et al., *Self-radiolysis of tritiated water. 3. The [radical dot]OH scavenging effect of bromide ions on the yield of H2O2 in the radiolysis of water by 60Co [gamma]-rays and tritium [small beta]-particles at room temperature.* RSC Advances, 2014. **4**(82): p. 43572-43581.
6. Butarbutar, S.L., et al., *Self-radiolysis of tritiated water. 2. Density dependence of the yields of primary species formed in the radiolysis of supercritical water by tritium [small beta]-particles at 400 [degree]C.* RSC Advances, 2014. **4**(44): p. 22980-22988.
7. Sunder, S., et al., *Oxidation of UO2 fuel by the products of gamma radiolysis of water.* Journal of Nuclear Materials, 1992. **190**: p. 78-86.

8. Sunder, S., D.W. Shoesmith, and N.H. Miller, *Oxidation and dissolution of nuclear fuel (UO2) by the products of the alpha radiolysis of water.* Journal of Nuclear Materials, 1997. **244**(1): p. 66-74.
9. Sunder, S., N.H. Miller, and D.W. Shoesmith, *Corrosion of uranium dioxide in hydrogen peroxide solutions.* Corrosion Science, 2004. **46**(5): p. 1095-1111.
10. Christensen, H., S. Sunder, and D.W. Shoesmith, *Oxidation of nuclear fuel (UO2) by the products of water radiolysis: development of a kinetic model.* Journal of Alloys and Compounds, 1994. **213**: p. 93-99.
12. EC Buck, RS Wittman, Radiolysis model formulation for integration with the mixed potential model, Pacific Northwest National Laboratory Report, July 2014, PNNL-23459.
13. EC Buck, RS Wittman, FN Skomurski, KJ Cantrell, BK McNamara, CZ Soderquist, Radiolysis Process Model, Pacific Northwest National Laboratory Report, July 2012, PNNL-21554.
